# Integrating Bioinformatics Tools Into Inquiry-Based Molecular Biology Laboratory Education Modules

**DOI:** 10.3389/feduc.2021.711403

**Published:** 2021-07-28

**Authors:** Carlos C. Goller, Melissa C. Srougi, Stefanie H. Chen, Laura R. Schenkman, Robert M. Kelly

**Affiliations:** 1 Biotechnology (BIT) Program, North Carolina State University, Raleigh, NC, United States,; 2 Department of Biological Sciences, College of Sciences, North Carolina State University, Raleigh, NC, United States,; 3 Department of Molecular Biomedical Sciences, College of Veterinary Medicine, North Carolina State University, Raleigh, NC, United States,; 4 Department of Chemical and Biomolecular Engineering, North Carolina State University, Raleigh, NC, United States

**Keywords:** bioinformatics, science education, molecular biotechnology, software tools, case studies

## Abstract

The accelerating expansion of online bioinformatics tools has profoundly impacted molecular biology, with such tools becoming integral to the modern life sciences. As a result, molecular biology laboratory education must train students to leverage bioinformatics in meaningful ways to be prepared for a spectrum of careers. Institutions of higher learning can benefit from a flexible and dynamic instructional paradigm that blends up-to-date bioinformatics training with best practices in molecular biology laboratory pedagogy. At North Carolina State University, the campus-wide interdisciplinary Biotechnology (BIT) Program has developed cutting-edge, flexible, inquiry-based Molecular Biology Laboratory Education Modules (MBLEMs). MBLEMs incorporate relevant online bioinformatics tools using evidenced-based pedagogical practices and in alignment with national learning frameworks. Students in MBLEMs engage in the most recent experimental developments in modern biology (e.g., CRISPR, metagenomics) through the strategic use of bioinformatics, in combination with wet-lab experiments, to address research questions. MBLEMs are flexible educational units that provide a menu of inquiry-based laboratory exercises that can be used as complete courses or as parts of existing courses. As such, MBLEMs are designed to serve as resources for institutions ranging from community colleges to research-intensive universities, involving a diverse range of learners. Herein, we describe this new paradigm for biology laboratory education that embraces bioinformatics as a critical component of inquiry-based learning for undergraduate and graduate students representing the life sciences, the physical sciences, and engineering.

## INTRODUCTION

Students benefit from inquiry-based laboratory courses (Myers and Burgess, 2003; Wallace et al., 2003; Weaver et al., 2008) and several initiatives have formed to help instructors implement these laboratory experiences in undergraduate and graduate curricula [CUR (CUR, 2021), CUREnet (CUREnet, 2021), etc.]. However, transitioning a lab course to the inquiry-based format requires an extensive commitment of time and resources. To lower the entry barrier for participation, pre-structured labs, with open research questions, could be centrally produced and distributed.

The Biotechnology Program (BIT) at North Carolina State University (NC State) (www.ncsu.edu/biotechnology) offers cutting-edge laboratory experiences for students coming from eight colleges. In addition to full-time teaching faculty, teaching postdoctoral scholars (Chen and Goller, 2019) are critical to the Program. As part of their training, teaching postdocs design, implement, and assess novel laboratory courses, based on their research expertise, ranging from metagenomics to protein sciences to organoids. These Molecular Biology Laboratory Education Modules (MBLEMs) are implemented through several iterations, and course details are subsequently published in relevant education journals. This paradigm exposes students to the latest in the biomolecular sciences, including hands-on training in cutting-edge techniques in the context of research questions, while allowing postdoctoral scholars to gain valuable teaching experience as well as develop their research agenda.

MBLEM creation involves iteration of a procedure we define as the “5D Process”: Designation, Design, Development, Deployment, and Dissemination (Supplementary Figure S1). After a topic has been Designated (i.e. committed to), approximately 1 year is taken for Design and Development of the course, with the instructor concomitantly participating in Deploying existing MBLEMs to understand the framework and possibilities. This Design includes “backwards design” (Wiggins and McTighe, 1998) of course structure and assessments from Designated learning outcomes and Development includes piloting of the proposed experiments and assessments, often by undergraduate student researchers during the academic year or summer months. The course is then Deployed by the instructor and a graduate teaching assistant for a small cohort (typically twelve students). Depending on the results of the Deployment, additional Design and Development, followed by another round of Deployment, may be needed before Dissemination, which includes publication in an appropriate educational journal. Reasons for additional rounds of Development include activities not working as intended, or assessments revealing that scaffolding of the material was insufficient to achieve the learning objectives. Through the 5D process, MBLEMs have been created and disseminated over the years (Witherow and Carson, 2011; Srougi and Carson, 2013; Ott and Carson, 2014; Lentz et al., 2017; Chen and Goller, 2020; Goller and Ott, 2020; Samsa et al., 2020; Garcia et al., 2021), ranging in topics from protein-protein interactions, signal transduction to metagenomics. This model has proven effective as demonstrated by the successful achievement of student learning outcomes and technical skills acquisition in these courses as assessed from quantitative course data (i.e., lab reports, exams, lab notebooks, and projects) and through analysis of qualitative student survey data.

## MBLEMS IN FOCUS

**FIGURE 1 |:**
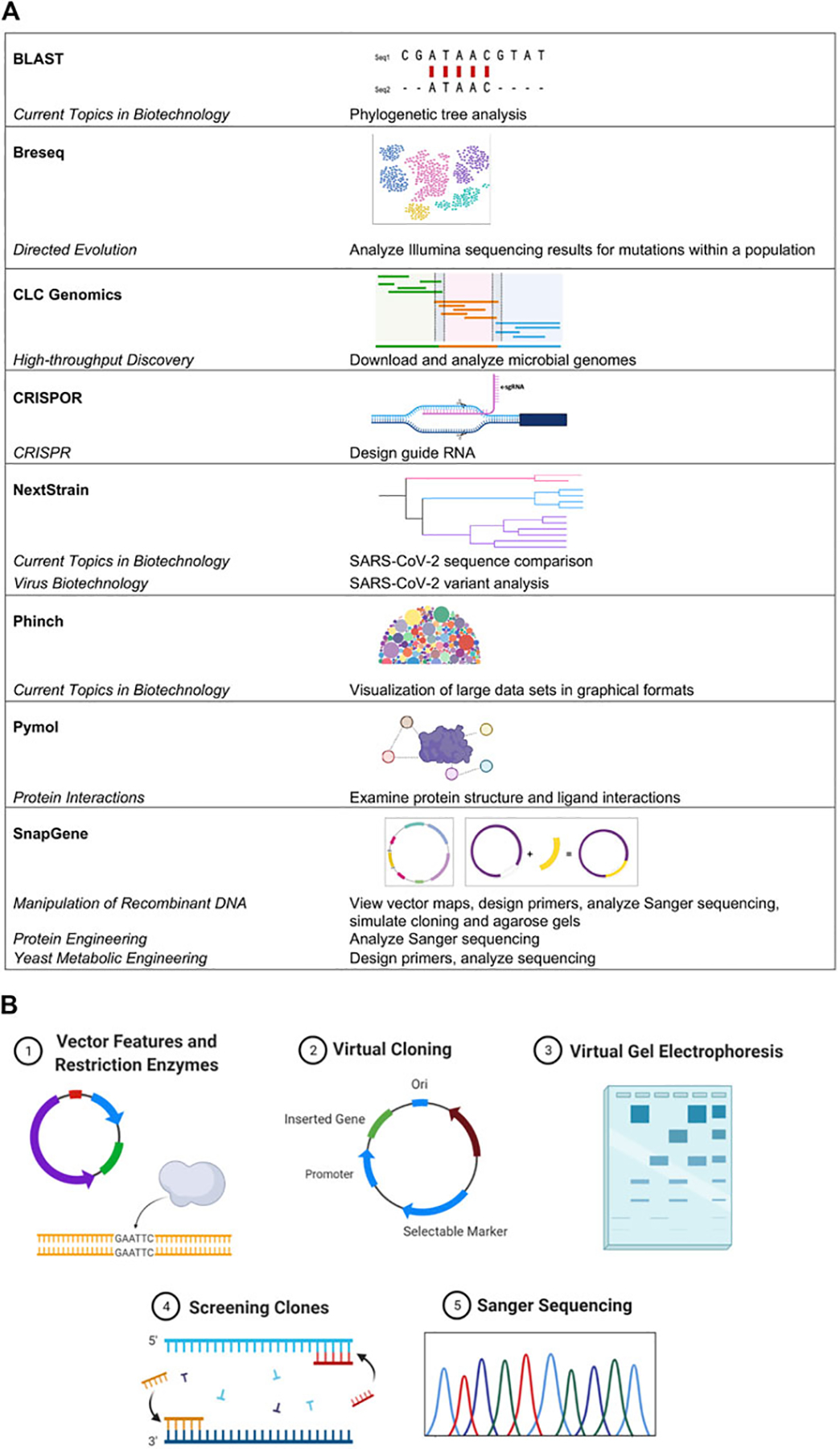
Bioinformatics usage across MBLEMs. **(A)** Examples of bioinformatics software used in MBLEMs. **(B)** Student-driven in silico cloning projects challenge individuals to practice using bioinformatics software to solve tasks they would encounter in molecular biology. Key aspects of the project design are illustrated including: vector features and restriction enzymes, virtual cloning, virtual gel electrophoresis, screening, and Sanger sequencing. Images created with BioRender.com.

The BIT Program serves students, ranging from freshmen that are not STEM majors to upper-level undergraduates and graduate students seeking life science-related degrees. MBLEMs are continually being created and updated to reflect current biotechnological advances and high impact teaching practices (HIP) (White, 2018). Bioinformatics are at the core of several of our course offerings and are included to varying degrees in all MBLEMs (Figure 1A), providing both vertical and horizontal integration of bioinformatics throughout the entire Program. Selected MBLEMs below illustrate how bioinformatics can be incorporated in the context of different class settings at varying educational levels.

## INTEGRATING BIOINFORMATICS INTO A FIRST-YEAR COURSE FOR STEM AND NON-STEM MAJORS

### Course Summary: Current Topics in Biotechnology

- 16-weeks, 4-credit lecture/laboratory course (twice weekly meetings of 2 h 45 min)
- General elective in the natural sciences for first-year students
- Introduction to the science and ethics of biotechnology

Current Topics in Biotechnology is a first-year inquiry lecture/laboratory course that provides science, technology, engineering, and mathematics (STEM) majors and non-STEM majors the opportunity to learn about biotechnology topics, ranging from biofuel production to genome editing using CRISPR-Cas9. The course has three goals: 1) “Think and Do” biotechnology, 2) Communicating scientific findings, and 3) Becoming a responsible community scientist. Learning outcomes for each of these goals are listed in Table 1.

It is challenging to teach bioinformatics to first-year students, especially those who are non-STEM majors. Introducing bioinformatics tools through the use of case studies is an effective way to involve all students. Different student interpretations of datasets provide a backdrop for rich discussions. Resources are available for peer-reviewed case studies on a variety of topics [e.g., CourseSource (Rosenwald et al., 2017), National Center for case Study Teaching in Science (NCCSTS, 2021)], thereby reducing the preparation required. A few examples of how we use bioinformatics in a first-year course are described below.

### Case Study in Metagenomics

Students are led through the case study Unique Down to Our Microbes (Lentz et al., 2017), exposing undergraduates to methodologies used to study various types of microbial life that inhabit human bellybuttons, while allowing agency in the direction of research inquiry. Students analyze community-science collected data using the open-access bioinformatics tool Phinch.org (Bik and Pitch Interactive Inc, 2014), an open-source tool for visualizing analyzing large open-source biological datasets (Figure 1A). With this software, students analyze a large cohort of data collected from individuals from around the world (Hulcr et al., 2012) to investigate trends in microbial biodiversity in the human belly button. The familiarity (and somewhat whimsical nature) of the bellybutton generates genuine curiosity: How often should one wash their own bellybutton? Does frequency affect their microbial biota? Students eagerly form hypotheses and share their data analysis with the class.

### Case Study in Virus Biotechnology

During the virus biotechnology unit, students use a case study to examine the spread and monitor the evolving genomes of SARS-CoV-2 in COVID-19: Where did you come from, where did you go? (Chen et al., 2020). Students use bioinformatics tools in the National Center for Biotechnology Information (NCBI) to perform sequence searches and alignments. Specifically, they learn how to interpret BLAST results (max score, query cover, E-value, and percent identity) and create phylogenetic trees of coronavirus sequence divergence using the open-source software Nextstrain (Hadfield et al., 2018). The website enables users to track real-time data of evolving pathogen populations and create interactive data visualizations. Data analyzed from NCBI BLAST and Nextstrain are used to answer important questions regarding SARS-CoV-2 spread. This case has been used widely in high school and college classrooms to engage students directly with the data underlying the COVID-19 pandemic, and can be adapted to answer emerging questions, such as the nature of the SARS-CoV-2 variants (Figure 1A).

## INTEGRATING BIOINFORMATICS IN A DUAL-LEVEL UNDERGRADUATE, GRADUATE COURSE IN MOLECULAR BIOTECHNOLOGY

### Course Summary: Manipulation of Recombinant DNA

- 16-weeks 4-credit lecture/laboratory course (lecture 1 h 50 min, lab 5 h weekly)
- Elective for upper-level undergraduate and graduate students
- Students perform a cloning project from gene to protein expression and testing

Manipulation of Recombinant DNA is a foundational lecture/lab course offered to STEM undergraduate majors and graduate students covering basic techniques in cloning, protein expression/purification, and prokaryotic and eukaryotic expression systems. Student learning objectives and assessments are described in Table 1. Lecture topics are directly related to the project-based laboratory sequence. For example, students learn about different types of screening methods and then perform three of those methods (i.e., restriction digestion, PCR screening, Sanger sequencing) (Garcia et al., 2021).

Bioinformatics tools in this course are first introduced through active learning assignments performed in collaborative groups, which we have found to be the most effective for student learning (Srougi et al., 2013; Tanner, 2017). Peer interactions are critical for learning key concepts and provide valuable experience in working in diverse groups, a critical skill in the biotechnology workforce. Introducing bioinformatics tools is a natural progression of the active-learning-based structure already established in the course. To extrapolate and hone in on their skills, students then individually complete a final capstone cloning project that involves bioinformatics. Since any software will involve a learning curve for students, it is imperative to develop students’ familiarity with bioinformatics programs through frequent use. This ensures that students can focus critically on the project itself without the burden of mastering new software. A brief description of how bioinformatics tools are used in this course can be found below:

### Molecular Cloning Visualized

Bioinformatics tools are introduced at the start of the course and interwoven throughout the curriculum. SnapGene software is the primary tool utilized to facilitate students’ understanding of gene cloning by allowing them to design primers, generate the results of Gibson assembly or ligation cloning, and simulate polymerase chain reaction (PCR), restriction digestion, and agarose gel electrophoresis (Figure 1B). Using this software, students reinforce hands-on laboratory skills virtually by simulating the course cloning project. Graduate and honors students in the course go on to utilize SnapGene in a capstone project where they design an experimental cloning and protein expression strategy for a gene of interest related to their own interests. Due to its extensive utility in simulating experiments in a visually appealing manner before any reagents or time are wasted in the lab, students typically continue to use this software outside of the classroom in their own research.

### Exposure to Complex Data Sets

Students in modern biotechnology education courses should have exposure to and practice with using complex data sets. To achieve this goal, the course incorporates the use of the La Cuadrilla case study (discussed in detail later). The case study presents a real-life scenario where villagers in La Cuadrilla, Mexico were getting sick from an unknown biological agent. Scientists from the Centers for Disease Control (CDC) collected water samples to perform a culture-independent diagnostic test with high-throughput sequencing. Students are given the raw data from this sequencing analysis and employ CLC Genomics software (Qiagen) to trim, filter, and analyze reads. CLC Genomics is an all-in-one software that enables analysis and visualization of data from all major next-generation sequencing (NGS) applications and provides an adaptable workflow for users depending upon their needs. The software does come at a fee, but discounted licenses are available for teaching purposes (see freeware options in Supplemental Table S1).

## BIOINFORMATICS IN 8-WEEK SPECIALTY COURSES

Upon completion of the foundational course in the Manipulation of Recombinant DNA, students can choose from a menu of continuously updated 8-week, 2-credit hour specialty module courses to complete the biotechnology minor. Each of these lecture/laboratory courses focus on a particular cutting-edge aspect of biotechnology with an emphasis on hands-on laboratory skills. Current examples include: Plant Genetic Engineering, CRISPR, High-Throughput Discovery, among others (see www.ncsu.edu/biotechnology). The selection of courses continually adapts with developments in the life sciences.

### Course Summary: Metagenomics

- 8-weeks lecture/laboratory course (1 h 50 min lecture, 5 h lab weekly)
- Pre-requisite is Manipulation of Recombinant DNA or equivalent
- Students analyze metagenomic populations from various niches

Metagenomics (Goller and Ott, 2020) is an inquiry-based course that provides advanced level undergraduate and graduate students hands-on exposure to a wide-variety of methods for analyzing unique and complex microbial communities. A focus is on computational skills to evaluate interactions between microbial populations and their surroundings. The course incorporates a variety of pedagogical practices to aid student learning in the process-orientated nature of metagenomics research. To this end, the course was built around concept mapping (Ritchhart et al., 2011) and reflective writing assignments. In the laboratory, students are guided through a series of wet labs where they isolated and purified genomic DNA, then made DNA libraries for NGS sequencing. During sequencing, students are introduced to bioinformatics tools, including the CyVerse Discovery Environment (Cyverse, 2021) and QIIME (Bolyen et al., 2019), to prepare them to perform data Q/C and analyses. Through this process, students are introduced to computing resources on the cloud. During the COVID-19 pandemic, this course was successfully delivered online asynchronously; the laboratory component in this context consisted of bioinformatics exercises, including data analysis case studies and publications using high-throughput approaches to understand microbial communities. The student learning objectives and assessments for Metagenomics are detailed in Table 1.

Bioinformatics are at the core of Metagenomics; highlights of the bioinformatics tools and activities used in Metagenomics are featured below.

## METAGENOMICS (TOOL) BOX

Interpreting microbial genomics with CLC. CLC Genomics Workbench (QIAGEN, 2021) and the Microbial Genomics Module (MGM) provide a powerful yet easy-to-use graphical interface through which students can import raw reads from an experiment they conduct in lab. Using the QIAGEN 16S/ITS panel and phased primers, sequencing libraries targeting multiple regions of the 16S ribosomal RNA gene and ITS can be prepared. CLC has a metagenomics workflow that is, included in the MGM that starts with importing and pairing reads and continues on to quality filtering and taxonomic classification using a downloaded database of choice (e.g., SILVA). CLC then displays taxonomic classification based on the sample metadata provided, allowing students to filter by sample type, re-graph by relative abundance, and run PERMANOVA analyses. This activity provides a user-friendly introduction used in the Metagenomics and Manipulation of Recombinant DNA MBLEMs.

CLC is used to engage students in analyzing next-generation sequencing datasets from a student-produced metagenomic survey. Using a case study approach (Herreid, 2011), students in the Manipulation of Recombinant DNA MBLEM learn about the use of high-throughput sequence to investigate an outbreak in a small village in rural central Mexico. The “La Cuadrilla” case study (Supplementary Table S2) challenges groups of students to analyze raw data and interpret it to make recommendations to the health department. A CLC metagenomics workflow that is, part of the MGM plugin of CLC enables students to work together to solve this mystery. This case study aligns with course learning objectives and introduces the use of databases, thus building on previous concepts addressed in the course. We created a second case study that addresses the concepts of genome assembly by having students assemble and explore sequencing results from a mystery yeast (Supplementary Table S2). While the cost of CLC can be significant, alternatives include free web-based platforms like Nephele (Weber et al., 2018), PUMAA (Mitchell et al., 2020), and KBase (Arkin et al., 2018). Nephele and KBase are important tools used in various MBLEMs to teach students about the analysis and applications of high-throughput sequencing. The importance of Nephele and KBase in MBLEMs is described below.

Nephele (Weber et al., 2018) is a web-based microbiome analysis pipeline developed by US National Institutes of Health National Institute of Allergy and Infectious Diseases. Nephele has pipelines for analysis of microbiome sequence data using popular tools, such as DADA2, QIIME2, and bioBakery. Users can upload datasets and modify workflow parameters to submit jobs for the heavy computational analyses. Results are then emailed with access to download results that include graphs, analyses, raw and filtered data, and log files. Students use Nephele to learn about the effects of different parameters and pipelines without the need of background in coding. For example, data from published microbiome studies can be reanalyzed to teach students about the significance of quality control and parameter optimization. Participants can also learn the importance of metadata for downstream analysis including hypothesis testing.

The KBase (Arkin et al., 2018) web-based platform developed by the US Department of Energy is used to analyze metagenomic datasets using sharable interactive workflow “narratives” focusing on metagenomic assembly, taxonomic identification, binning, and metabolic modeling. Students learn about “read hygiene” and compare different assemblers and the advantages and limitations of short-read taxonomic inferences and contig binning. Students employ narratives to learn key steps and explore new datasets, learning key concepts, and procedures in the process of examining datasets.

QIIME2/DADA2 (Bolyen et al., 2019) is used to expose participants to high-performance computing (HPC) and the ability to submit different jobs. Students log in and complete the QIIME Moving Pictures tutorial to practice working in the command-line environment and understand the fundamental steps in a 16S amplicon metagenomics analysis. Emphasis is placed on the use of DADA2 and the differences between Operational Taxonomic Units (OTUs) and Amplicon Sequence Variants (ASVs) in the context of accuracy and reproducibility. Students use a common script to run tutorials and then adapt it for their own datasets.

SnapGene (Science, 2021) bioinformatics software has been integrated across several BIT MBLEMs to help students understand critical molecular biology concepts and gain experience with sequence analysis tools and approaches. This has been done in alignment with course objectives and frameworks describing bioinformatics core competencies for undergraduate education (Sayres et al., 2018; Williams et al., 2019). The flagship MBLEM Manipulation of Recombinant DNA includes activities using SnapGene, as discussed above. Additionally, SnapGene has been vertically integrated in other MBLEMs; in Yeast Metabolic Engineering SnapGene is used to analyze sequencing reactions and identify barcode sequences from promising yeast mutants producing beta-carotene. Students learn the concepts and skills using user-friendly software to apply in more advanced MBLEMs (Figure 1B). In addition, SnapGene integrates with the electronic lab notebook (ELN) system we use in the laboratory for several MBLEMs (LabArchives).

While SnapGene is not free, a site license can be purchased for campus-wide use; as an alternative, freeware sequence visualization, and analysis software such as Benchling and ApE are available alternatives (Supplementary Table S1). Besides cost, instructors should consider the accessibility features of the software and logistics required to provide access to students. For example, during the pandemic, students had to access SnapGene off-campus using full tunnel VPN, which required an additional setup on the part of the users.

## SUMMARY

The BIT Program at NC State offers a dynamic set of cutting-edge courses in modern biotechnology through an innovative paradigm. A special feature of MBLEM design is the incorporation of datasets and bioinformatics tools into each course offering, based on best pedagogical practices (i.e., user-friendly software, peer support, and authentic inquiry-based projects). The examples provided encourage students to perform novel analyses on real datasets. Currently, MBLEMs are being actively employed by a wide variety of institutional partners to demonstrate that bioinformatics modules are portable and valuable in supplementing molecular biotechnology training.

## Supplementary Material

Figure 1

Table 1

Table 2

## Figures and Tables

**TABLE 1 |:** MBLEM course objectives and assessment methods.

MBLEM: Current topics in biotechnology	
Course objectives (COs)	Methods for assessing COs
By the end of this course, students will be able to	
CO 1: “Think and do” biotechnology	
Understand the scientific concepts that underlie and experiment Generate testable hypotheses Create figures ot scientific results Interpret qualitative and quantitative experimental results	Lab activities Lab reports Guided worksheets Quizzes
CO 2: Communicate scientific findings	
Keep detailed, concise, organized record of scientific experiments Communicate experimental results in a discipline-appropriate writing Explain biotechnology concepts to different audiences	Lab notebook entries Lab reports Multimedia presentations
CO 3: Become a responsible community scientist	
Identify and critique biotechnology issues relating to society or the responsible conduct of biotechnology research	Reflection journal Multimedia presentations
MBLEM: Manipulation of recombinant DNA	
CO 1: Design experiments to manipulate DNA	Lab reports
Design strategies to manipulate DNAto create new proteins	Lab reports Take home exams Individual exams
Perform a variety of techniques in the manipulation of recombinant DNA and protein expression	Final exam BIT 510 project Active learning activities
CO 2: Communicate scientific findings	
Create a detailed written record of experimental procedures, results, and conclusions Interpret data and controls related to gene cloning, protein expression and hypothesis testing Troubleshoot experiments that do not work Reflect on their own thinking and the thinking of others	Lab notebook Lab reports Active learning activities
CO 3: Evaluate research questions	
Evaluate a specific hypothesis	Lab notebook Lab reports Take home exams Final exam
CO 4: Exercise problem-solving skills in molecular biotechnology	
Apply critical and creative thinking skills and behaviors in the process of solving problems or addressing questions	Take home exams Individual exams Lab report 3 Final exam
MBLEM: Metagenomics	
CO 1: Become a responsible community scientist	
Demonstrate laboratory skills required of a modern-day molecular biologist m the era of next-generation sequencing. This includes keeping detailed and accurate laboratory notes (e.g., electronic records for sequence analyses) and choosing appropriate sequencing based on goals	Critical thinking scenarios and discussion posts
CO 2: Bead scientific literature	
Read a scientific article and evaluate how bioinformatics methods were employed by the authors to explore a particular hypothesis. (From CourseSource framework]	Article summaries and annotations Collaborative notes Knowledge check questions Individual podcast explanation assignment
CO 3: Evaluate research questions	
Given a scientific question, develop a hypothesis and define computational approaches that could be used to explore the hypothesis. (From CourseSource framework)	Group data analyses project drafts and final submission
CO 4: Analyze experimental data	
Use pre-existing tools to analyze a metagenomic data set to determine the set of organisms present in a metagenomic sample (e.g., 16s rRNA, greengenes, mothur, etc.). (From CourseSource framework)	Individual podcast explanation assignment case studies using KBase and QIIME2/DADA2
CO 5: Critically evaluate limitations of data analysis	
Interpret data and identify limitations related to metagenomic surveys	Individual podcast explanation assignment Group data analyses project drafts and final submission
CO 6: (For graduate students) Explain analyses to different audiences	
Design a critical thinking scenario and explain analyses for hypothesis testing of metagenomic data	Video tutorial

## Data Availability

The original contributions presented in the study are included in the article/Supplementary Material, further inquiries can be directed to the corresponding author.
