# Supplementary material for: Integrating Bioinformatics Tools Into Inquiry-Based Molecular Biology Laboratory Education Modules
Source: Front Educ (Lausanne). Author manuscript; Available in PMC 2022 Jan 13. (PMC8758113; doi:10.3389/feduc.2021.711403)
Supplement: Figure 1 [file NIHMS1769968-supplement-Figure_1.pdf]

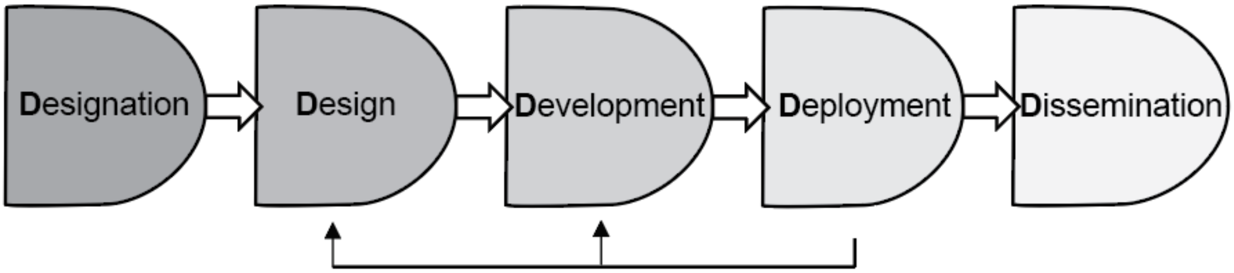

Supplementary Figure 1: **The 5D process of MBLEM Development**. After topic Designation, instructors spend about a year working on Design and Development, followed by Deployment in the laboratory or online. After initial Deployment, further Design and Development may be needed prior to Dissemination. The 5D process is dynamic: using evidence from evaluation and implementation at different partner institutions, MBLEMs are continuously updated and improved.
