# Supplementary material for: Integrating Bioinformatics Tools Into Inquiry-Based Molecular Biology Laboratory Education Modules
Source: Front Educ (Lausanne). Author manuscript; Available in PMC 2022 Jan 13. (PMC8758113; doi:10.3389/feduc.2021.711403)
Supplement: Table 1 [file NIHMS1769968-supplement-Table_1.pdf]

**Supplementary Table 1.** List of Freeware for Sequence Visualization and Cloning.	
**Software**	**Website**
ApE	https://jorgensen.biology.utah.edu/wayned/ape/
Benchling	https://www.benchling.com/
SerialCloner	http://serialbasics.free.fr/Serial_Cloner.html
UGENE	http://ugene.net/
