# Supplementary material for: Integrating Bioinformatics Tools Into Inquiry-Based Molecular Biology Laboratory Education Modules
Source: Front Educ (Lausanne). Author manuscript; Available in PMC 2022 Jan 13. (PMC8758113; doi:10.3389/feduc.2021.711403)
Supplement: Table 2 [file NIHMS1769968-supplement-Table_2.pdf]

**Supplementary Table 2.** Mystery Yeast and La Cuadrilla Case Study Learning Objectives and Workflow.		
**Case Study**	**La Cuadrilla Outbreak**	**Mystery Yeast Investigation**
Learning Objectives	• **Compute** summary statistics for the provided FASTQ files.<br>• **Apply** CLC tools to trim and filter reads.<br>• **Apply** the CLC Microbial Genomics Module (MGM) Data QC and Taxonomic Profiling tools to process sequence reads.<br>• **Explain** how you can identify different species in a community using next-generation or high-throughput sequencing.<br>• **Evaluate** data from a metagenomics case study (case study scenario and data analyses with CLC).	• **Compare** and **contrast** Sanger DNA sequencing and next-generation or high-throughput sequencing.<br>• **Compute** summary statistics for the provided FASTQ files.<br>• **Apply** CLC tools to trim and filter reads.<br>• **Evaluate** data from a case study (case study scenario and data analyses with CLC).
Format	Group	Individual or Group
Concepts	• FASTQ files and sequence quality trimming/filtering<br>• Metagenomics	• Compare and contrast Sanger and NGS<br>• Genome assembly<br>• Genome sequence scanning<br>• Using BLAST to identify sequences
